# Predictors of Ischemic Stroke in Patients With Non-valvular Atrial Fibrillation: A Retrospective Study

**DOI:** 10.7759/cureus.112522

**Published:** 2026-07-12

**Authors:** Muhammad Waleed Jabbar, Tayyaba Shabbir, Saad Binliaquat, Muzammil Abbas, Abdul Rehman Ahmad Akhtar, Mudassar Nisar, Somia Bibi

**Affiliations:** 1 Internal Medicine, Kharkiv National Medical University, Kharkiv, UKR; 2 Internal Medicine, Adel Care Medical, Buraidah, SAU; 3 Internal Medicine, Cardiology, Emergency Medicine, King Fahad Specialist Hospital, Buraidah, SAU; 4 Internal Medicine, Jinnah Hospital Lahore, Lahore, PAK; 5 Internal Medicine, Royal Derby Hospital, Derby, GBR; 6 Internal Medicine, Sahiwal Medical College, Sahiwal, PAK; 7 Internal Medicine, Allama Iqbal Medical College, Lahore, PAK; 8 Internal Medicine, George Eliot Hospital, Nuneaton, GBR; 9 Internal Medicine, Rawalpindi Medical University, Rawalpindi, PAK

**Keywords:** anticoagulation, atrial, atrial fibrillation, comorbidities, fibrillation, inadequate, ischemic, predictor, retrospective, stroke

## Abstract

Background

Atrial fibrillation (AF) is the most common sustained cardiac arrhythmia and a major cause of ischemic stroke (IS). Although the Congestive heart failure (or left ventricular dysfunction), Hypertension, Age ≥ 75 years, Diabetes mellitus, prior Stroke, transient ischemic attack (TIA), or thromboembolism, Vascular disease (prior heart attack, peripheral artery disease, or aortic plaque), Age 65 to 74 years, and ​​​​​​​Sex category (Female) or CHA₂DS₂-VASc score is widely used for stroke risk stratification, additional clinical, treatment-related, and echocardiographic factors may influence stroke risk. This study aimed to determine the frequency of IS and identify its independent predictors among patients with non-valvular AF.

Methods

This retrospective study included 350 patients with non-valvular AF treated at a tertiary care hospital in Rawalpindi, Pakistan. Demographic, clinical, anticoagulation, CHA₂DS₂-VASc, and echocardiographic data were extracted from medical records. Statistical analyses were performed using IBM SPSS Statistics for Windows, Version 25 (Released 2017; IBM Corp., Armonk, New York, United States). Group comparisons were conducted using the independent-samples t-test and chi-square test, followed by multivariable logistic regression to identify independent predictors of IS.

Results

From the total study population, 71 (20.28%) experienced IS. Patients with stroke had a significantly higher mean CHA₂DS₂-VASc score than those without stroke (4.50 ± 1.40 vs. 3.20 ± 1.20, p<0.001). On initial analysis, age ≥65 years, previous stroke/TIA, vascular disease, smoking, chronic kidney disease, inadequate anticoagulation, left atrial enlargement, reduced left ventricular ejection fraction (LVEF<40%), left atrial appendage (LAA) thrombus/spontaneous echo contrast (SEC), and CHA₂DS₂-VASc score ≥4 were significantly associated with IS (all p<0.05). Multivariable logistic regression confirmed age ≥65 years (adjusted odds ratio (AOR)=2.59; p=0.003), previous stroke/TIA (AOR=2.37; p=0.012), vascular disease (AOR=1.88; p=0.045), smoking (AOR=1.93; p=0.039), chronic kidney disease (AOR=2.12; p=0.028), inadequate anticoagulation (AOR=2.90; p=0.001), left atrial enlargement (AOR=2.32; p=0.007), reduced LVEF (AOR=1.94; p=0.042), and LAA thrombus/SEC (AOR=3.07; p=0.004) as independent predictors of IS.

Conclusion

IS was observed in a considerable proportion of patients with non-valvular AF. IS was independently associated with advanced age, previous stroke/TIA, vascular disease, smoking, chronic kidney disease, inadequate anticoagulation, left atrial enlargement, reduced LVEF, and LAA thrombus/SEC. Assessment of these factors, in addition to the CHA₂DS₂-VASc score, may enhance risk stratification and facilitate timely implementation of preventive strategies.

## Introduction

Atrial fibrillation (AF) is the most prevalent persistent heart rhythm abnormality globally. The prevalence of AF has risen considerably worldwide, increasing from an estimated 33.5 million cases in 2010 to 59 million cases in 2019 [1]. However, the true burden of the disease may be even greater, as many individuals remain unaware of their condition due to asymptomatic presentation and inadequate screening opportunities. Public awareness regarding AF also remains suboptimal, with only 48% of the global population able to identify arrhythmia and its associated complications [2].

The prevalence of AF continues to rise with population aging and the increasing burden of cardiovascular comorbidities, including hypertension, diabetes mellitus, heart failure, and vascular disease [3]. AF affects approximately 1%-2% of the global adult population, with its prevalence increasing markedly with advancing age, reaching 5%-10% among individuals aged 70-79 years, and as high as 17% in those aged 80 years and older [4]. Consequently, AF is associated with significant morbidity, mortality, frequent hospitalizations, and substantial healthcare burden [4,5].

Among the complications of AF, ischemic stroke (IS) represents the most serious and clinically significant outcome. Patients with AF have an approximately five-fold higher risk of IS than the general population [6]. Approximately one-quarter of IS are attributed to cardioembolism, with AF being the most common underlying etiology [3,7]. Furthermore, AF-related strokes are often associated with greater disability, higher recurrence rates, and increased mortality compared with strokes of other etiologies. Given the substantial individual and societal burden of stroke, accurate identification of patients at increased risk remains a cornerstone of AF management [8].

The pathophysiological link between AF and IS is multifactorial. Ineffective atrial contraction promotes blood stasis, particularly within the left atrial appendage (LAA), facilitating thrombus formation. Endothelial dysfunction, inflammation, platelet activation, and hypercoagulability further contribute to thrombogenesis. Structural and functional cardiac abnormalities, including left atrial enlargement, reduced left ventricular ejection fraction (LVEF), and LAA thrombus or spontaneous echo contrast (SEC), have also been associated with an increased risk of thromboembolic events [1,9-11].

The Congestive heart failure (or left ventricular dysfunction), Hypertension, Age≥ 75 years, Diabetes mellitus, ​​​​​​​prior Stroke, transient ischemic attack (TIA), or thromboembolism, ​​​​​​​Vascular disease (prior heart attack, peripheral artery disease, or aortic plaque), ​​​​​​​Age 65 to 74 years, and ​​​​​​​Sex category (Female) or CHA₂DS₂-VASc score is the most widely used tool for stroke risk stratification in patients with AF and incorporates established risk factors [6,12]. However, emerging evidence suggests that additional factors, including smoking, chronic kidney disease, inadequate anticoagulation, left atrial enlargement, reduced LVEF, and LAA abnormalities, may independently contribute to stroke risk beyond conventional risk prediction models [13-18].

Several studies conducted in various parts of the world have investigated predictors of IS among patients with AF and identified important associations with demographic characteristics, cardiovascular comorbidities, anticoagulation status, and echocardiographic abnormalities [6,7,17,18]. Although studies from Pakistan have also reported similar findings, they have primarily been conducted in individual tertiary care centers. Differences in patient demographics, cardiovascular risk profiles, healthcare delivery, and anticoagulation practices may influence the applicability and generalizability of these findings across different regions of the country [10,19]. Consequently, validation of these predictors in independent patient cohorts remains important.

Given the growing burden of AF and IS in Pakistan, further evaluation of stroke predictors in diverse clinical settings is essential to improve risk stratification, optimize anticoagulation strategies, and reduce preventable cerebrovascular events. Therefore, the primary objective of this study was to determine the frequency of IS among patients with non-valvular AF managed at a tertiary care hospital in Rawalpindi, Pakistan. The secondary objective was to identify the independent demographic, clinical, treatment-related, and echocardiographic predictors associated with IS in this population. By providing evidence from an independent patient cohort, this study complements the existing literature and contributes to a better understanding of stroke risk factors in the Pakistani population.

## Materials and methods

Study design and study population

This retrospective cohort study was conducted at Benazir Bhutto Hospital, Rawalpindi, Pakistan. Medical records of 350 patients with non-valvular AF diagnosed between January 2022 and January 2025 were retrospectively reviewed. The date of AF diagnosis was considered the index date. Baseline demographic, clinical, treatment-related, and echocardiographic variables recorded at the time of AF diagnosis were extracted from the medical records. Patients were subsequently followed for the occurrence of imaging-confirmed IS during the study period and were classified according to stroke status. Ethical approval was obtained from the Institutional Research and Ethics Committee (approval no. BBH.ERB.250/292) prior to data collection, and patient confidentiality was maintained throughout the study in accordance with institutional and international ethical standards.

Inclusion and exclusion criteria

All patients with documented non-valvular AF who met the study eligibility criteria were included. Patients aged 30 years or older with a confirmed diagnosis of non-valvular AF, complete baseline demographic, clinical, treatment-related, laboratory, and echocardiographic records, and documented follow-up for IS during the study period were eligible for inclusion. Patients with valvular AF, congenital heart disease, hemorrhagic stroke, active malignancy associated with a hypercoagulable state, or incomplete medical records that precluded assessment of study variables or outcomes were excluded from the study.

Primary and secondary outcomes

The primary outcome was the frequency of IS among patients with AF. The secondary outcome was the identification of clinical, demographic, and echocardiographic predictors associated with IS in patients with AF.

Data collection

AF was diagnosed according to established American Heart Association clinical guidelines, employing twelve-lead electrocardiography (ECG) [20]. The diagnosis of IS was confirmed through neuroimaging findings on computed tomography (CT) or magnetic resonance imaging (MRI), as interpreted by certified radiologists in accordance with American Stroke Association guidelines [21]. Anticoagulation status was assessed from medical records available after AF diagnosis and before the occurrence of IS. Inadequate anticoagulation was defined as subtherapeutic anticoagulation in warfarin users (international normalized ratio or INR <2.0) or documented non-adherence to direct oral anticoagulant (DOAC) therapy, as recorded before the stroke event [22]. Data were extracted from patients' medical records using a structured, self-designed proforma consisting of two sections. The first section captured baseline demographic and clinical information recorded at the time of AF diagnosis, including age, sex, previous history of stroke/TIA, heart failure, hypertension, diabetes mellitus, vascular disease, smoking status, chronic kidney disease, anticoagulation status, and the CHA₂DS₂-VASc score. The second section comprised baseline echocardiographic findings extracted from available echocardiography reports in the medical records, including left atrial enlargement (left atrial diameter >40 mm), reduced LVEF (<40%), and the presence of LAA thrombus or SEC. Owing to the retrospective study design, the specific echocardiographic modality (transthoracic or transesophageal echocardiography) was not consistently documented [23,24]. The occurrence of IS during follow-up was confirmed by CT or MRI, as documented in the medical records.

Data analysis

Statistical evaluation of data was performed with IBM SPSS Statistics version 25 (Released 2017; IBM Corp., Armonk, New York, United States). The normality of continuous variables was assessed using the Shapiro-Wilk test. Continuous variables were reported as mean ± standard deviation, whereas categorical variables were summarized as frequencies and percentages. Differences between patients with and without IS were assessed using the independent-samples t-test for continuous variables and the Chi-square test for categorical variables. Variables demonstrating statistical significance in the univariate analysis (p<0.05), together with clinically relevant variables supported by previous literature, were entered into the multivariable logistic regression model to identify independent predictors of IS among patients with AF. Model calibration of the multivariable logistic regression model was assessed using the Hosmer-Lemeshow goodness-of-fit test. A p-value>0.05 was considered indicative of adequate agreement between observed and predicted probabilities. Statistical significance was defined as a p-value≤0.05.

## Results

A total of 350 patients with non-valvular AF were included in the study. IS occurred in 71 (20.28%) patients, whereas 279 (79.72%) had no IS. The overall mean age was 68.42 ± 15.80 years, and the mean CHA₂DS₂-VASc score was 3.40 ± 1.50. Patients with IS were significantly older than those without stroke (73.78 ± 10.39 vs. 62.15 ± 10.72 years, p<0.001) and had a significantly higher mean CHA₂DS₂-VASc score (4.50 ± 1.40 vs. 3.20 ± 1.20, p<0.001). Likewise, a CHA₂DS₂-VASc score ≥4 was significantly more common among patients with IS than among those without stroke (77.47% vs. 40.14%, p<0.001).

Table 1 demonstrates that IS was significantly associated with age ≥65 years, previous stroke/TIA, vascular disease, smoking, chronic kidney disease, inadequate anticoagulation, left atrial enlargement, reduced LVEF, LAA thrombus/SEC, and a CHA₂DS₂-VASc score ≥4.

**Table 1:** Baseline characteristics of patients with atrial fibrillation stratified by ischemic stroke status TIA: Transient ischemic attack; LVEF: Left ventricular ejection fraction; LAA: Left atrial appendage; SEC: Spontaneous echo contrast.

Variable	Category	Stroke (n=71)	No stroke (n=279)	Test Statistic (χ²)	p-value
Age	≥65 years	49 (69.00%)	103 (36.91%)	22.78	<0.001
	<65 years	22 (31.00%)	176 (63.09%)		
Gender	Male	43 (60.56%)	158 (56.63%)	0.36	0.548
	Female	28 (39.44%)	121 (43.37%)		
Previous stroke/TIA	Yes	24 (33.80%)	40 (14.33%)	13.14	<0.001
	No	47 (66.20%)	239 (85.67%)		
Heart failure	Yes	19 (26.76%)	55 (19.71%)	1.69	0.194
	No	52 (73.24%)	224 (80.29%)		
Hypertension	Yes	49 (69.01%)	171 (61.29%)	1.45	0.228
	No	22 (30.99%)	108 (38.71%)		
Diabetes mellitus	Yes	33 (46.48%)	108 (38.71%)	1.37	0.242
	No	38 (53.52%)	171 (61.29%)		
Vascular disease	Yes	27 (38.02%)	61 (21.86%)	7.59	0.006
	No	44 (61.98%)	218 (78.14%)		
Dyslipidemia	Yes	31 (43.66%)	99 (35.48%)	1.58	0.209
	No	40 (56.34%)	180 (64.52%)		
Smoking	Yes	29 (40.84%)	66 (23.65%)	8.05	0.005
	No	42 (59.16%)	213 (76.35%)		
Chronic kidney disease	Yes	22 (30.98%)	42 (15.06%)	9.74	0.002
	No	49 (69.02%)	237 (84.94%)		
Inadequate anticoagulation	Yes	36 (50.70%)	67 (24.02%)	18.06	<0.001
	No	35 (49.30%)	212 (75.98%)		
Left atrial enlargement	Yes	39 (54.92%)	91 (32.61%)	11.31	0.001
	No	32 (45.08%)	188 (67.39%)		
Reduced LVEF	Yes	26 (36.61%)	60 (21.51%)	6.85	0.009
	No	45 (63.39%)	219 (78.49%)		
LAA thrombus/SEC	Yes	18 (25.35%)	27 (9.67%)	12.42	<0.001
	No	53 (74.65%)	252 (90.33%)		
CHA₂DS₂-VASc ≥4	Yes	55 (77.47%)	112 (40.14%)	30.14	<0.001
	No	16 (22.53%)	167 (59.86%)		

In contrast, gender, hypertension, diabetes mellitus, dyslipidemia, and heart failure showed no significant association with ischemic stroke. 

Table 2 shows that age ≥65 years, previous stroke/TIA, vascular disease, smoking, chronic kidney disease, inadequate anticoagulation, left atrial enlargement, reduced LVEF, and LAA thrombus/ SEC remained independent predictors of IS among patients with AF.

**Table 2:** Multivariable logistic regression analysis of predictors of ischemic stroke among patients with atrial fibrillation OR: Odds ratio; CI: Confidence interval; TIA: Transient ischemic attack; LVEF: Left ventricular ejection fraction; LAA: Left atrial appendage; SEC: Spontaneous echo contrast.

Variable	Crude OR	95% CI	p-value	Adjusted OR	95% CI	p-value
Age ≥65 years	3.82	2.18–6.67	<0.001	2.59	1.40–4.80	0.003
Male gender	1.19	0.70–2.04	0.548	1.14	0.62–2.05	0.714
Previous stroke/TIA	3.07	1.66–5.60	<0.001	2.37	1.22–4.65	0.012
Heart failure	1.48	0.81–2.77	0.194	1.27	0.68–2.55	0.482
Hypertension	1.42	0.80–2.48	0.228	1.19	0.65–2.23	0.601
Diabetes mellitus	1.39	0.84–2.36	0.242	1.15	0.63–2.19	0.648
Vascular disease	2.18	1.26–3.89	0.006	1.88	1.05–3.48	0.045
Dyslipidemia	1.40	0.80–2.42	0.209	1.14	0.63–2.12	0.693
Smoking	2.24	1.30–3.87	0.005	1.93	1.04–3.57	0.039
Chronic kidney disease	2.55	1.39–4.72	0.002	2.12	1.10–4.15	0.028
Inadequate anticoagulation	3.27	1.87–5.62	<0.001	2.90	1.58–5.35	0.001
Left atrial enlargement	2.54	1.49–4.28	0.001	2.32	1.28–4.24	0.007
Reduced LVEF	2.12	1.15–3.77	0.009	1.94	1.03–3.75	0.042
LAA thrombus/SEC	3.15	1.61–6.28	<0.001	3.07	1.44–6.61	0.004

Among these, LAA thrombus/SEC and inadequate anticoagulation demonstrated the strongest associations with IS, whereas male gender, hypertension, diabetes mellitus, dyslipidemia, and heart failure were not independently associated with stroke occurrence.

## Discussion

The present study identified several independent predictors of IS among patients with non-valvular AF. Advanced age, previous stroke/TIA, vascular disease, smoking, chronic kidney disease, inadequate anticoagulation, left atrial enlargement, reduced LVEF, and LAA thrombus/SEC were independently associated with stroke occurrence. These findings suggest that stroke risk in AF extends beyond the traditional components of the CHA₂DS₂-VASc score and highlight the additional prognostic value of treatment-related and echocardiographic factors [10,15,16].

Advanced age was independently associated with IS, consistent with the existing literature demonstrating that aging promotes atrial fibrosis, endothelial dysfunction, vascular stiffness, and a prothrombotic state. Elderly patients also have a greater burden of cardiovascular comorbidities, further increasing thromboembolic risk [3,12].

A previous history of stroke or TIA was among the strongest independent predictors of IS. This may be attributed to persistent atrial thrombus formation, endothelial dysfunction, an underlying prothrombotic state, and treatment non-adherence resulting in inadequate anticoagulation, all of which increase the risk of recurrent cerebral embolism. Our findings are consistent with previous reports and further support the inclusion of prior stroke/TIA as a major component of the CHA₂DS₂-VASc score [8,12].

Vascular disease also emerged as an independent predictor of IS. The presence of vascular disease reflects widespread atherosclerosis and systemic endothelial injury, which contribute to plaque instability, increased platelet activation, and enhanced thromboembolic susceptibility. In patients with AF, these vascular abnormalities may act synergistically with blood stasis to promote thrombus formation and subsequent cerebral embolization. These findings are consistent with published evidence demonstrating an association between vascular disease and IS in patients with AF [6,9].

Smoking was another significant predictor of IS. Cigarette smoking induces endothelial injury, oxidative stress, platelet activation, chronic systemic inflammation, and accelerated atherosclerosis while impairing fibrinolysis. These pathophysiological changes create a hypercoagulable state that enhances thrombus formation and increases the likelihood of IS in patients with AF. A similar association between smoking and IS has been reported in the existing literature [13,19].

Similarly, chronic kidney disease was independently associated with IS in our study. Chronic kidney disease promotes endothelial dysfunction, chronic inflammation, oxidative stress, and activation of the coagulation cascade, resulting in a prothrombotic state that increases the risk of thrombus formation and cerebral embolism. These observations are supported by previous studies demonstrating an elevated thromboembolic risk among patients with AF and impaired renal function [14,15].

Inadequate anticoagulation demonstrated a strong independent association with IS, highlighting the importance of maintaining therapeutic anticoagulation. Poor INR control among warfarin users and non-adherence to DOACs reduce protection against thrombus formation and remain important challenges, particularly in resource-limited settings where regular monitoring may be difficult. Comparable findings have been reported in previous studies demonstrating that suboptimal anticoagulation substantially increases the risk of IS in patients with AF [10,17,18].

In addition to clinical and treatment-related factors, echocardiographic abnormalities also demonstrated significant prognostic value for IS. Among the echocardiographic variables, left atrial enlargement, reduced LVEF, and LAA thrombus/SEC emerged as significant independent predictors. Left atrial enlargement reflects advanced atrial remodeling and impaired mechanical function, predisposing to blood stasis and thrombus formation. Reduced LVEF contributes to impaired cardiac output and intracardiac blood stasis, further increasing thromboembolic risk. The presence of LAA thrombus or SEC represents direct evidence of intracardiac thrombogenesis and is considered one of the strongest imaging predictors of cardioembolic stroke. Comparable associations have been reported in previous echocardiographic studies, which consistently identified these structural and functional abnormalities as important predictors of IS in patients with AF [6,10,16].

Although hypertension, diabetes mellitus, dyslipidemia, heart failure, and gender were not independently associated with IS in the present study, the available literature remains inconsistent. While several studies have identified these factors as significant contributors to thromboembolic risk in patients with AF, others have similarly reported no independent association after adjustment for established clinical and echocardiographic predictors [6,12,14,19]. The absence of significance in our study may therefore reflect differences in study populations, sample size, or the stronger prognostic influence of variables such as previous stroke/TIA, inadequate anticoagulation, and adverse echocardiographic findings.

The significantly higher mean CHA₂DS₂-VASc score among patients with IS reinforces its established role in stroke risk stratification. However, the independent predictive value of chronic kidney disease, inadequate anticoagulation, smoking, and echocardiographic abnormalities suggests that stroke risk assessment may be enhanced by incorporating clinical, treatment-related, and imaging factors beyond the conventional CHA₂DS₂-VASc score [10,15,16].

Although previous studies, have evaluated predictors of IS in patients with AF, our study provides independent evidence from a different tertiary care center in Pakistan using a separate patient cohort. The consistency of our findings with previous reports strengthens the evidence supporting established clinical and echocardiographic predictors of IS and enhances their generalizability across different healthcare settings within the country [10,19]. Furthermore, our study incorporated a clearly defined temporal framework, with baseline variables documented before the occurrence of IS, thereby strengthening the interpretation of these variables as predictors rather than simple correlates.

From a clinical perspective, these findings emphasize the importance of a comprehensive risk assessment in patients with AF. In addition to routine CHA₂DS₂-VASc evaluation, clinicians should assess renal function, smoking status, anticoagulation adequacy, and echocardiographic parameters, particularly left atrial enlargement, reduced LVEF, and the presence of LAA thrombus or SEC. Early identification of these high-risk features may facilitate optimization of anticoagulation therapy, targeted modification of cardiovascular risk factors, and closer clinical surveillance, thereby reducing the risk of preventable IS.

This study has several limitations. First, its retrospective single-center design precludes causal inference and may limit the generalizability of the findings. Second, reliance on medical records may have introduced information bias, residual confounding, and potential selection bias due to the exclusion of patients with incomplete records. Third, because the specific echocardiographic modality (transthoracic or transesophageal echocardiography) was not consistently documented, variation in imaging technique may have influenced the detection of LAA thrombus and SEC, potentially introducing detection bias. Fourth, anticoagulation quality was assessed only by INR values and documented adherence, while interactions between the CHA₂DS₂-VASc score and additional predictors were not evaluated. Finally, the absence of long-term follow-up precluded assessment of future stroke risk. Therefore, prospective multicenter cohort studies with standardized echocardiographic protocols, comprehensive assessment of anticoagulation quality, and external validation in diverse populations are warranted to confirm and extend these findings.

Despite these limitations, this study provides important real-world evidence regarding predictors of IS among patients with non-valvular AF in Pakistan. Beyond the conventional CHA₂DS₂-VASc score, our findings demonstrate that treatment-related factors, particularly inadequate anticoagulation, together with chronic kidney disease, smoking, and adverse echocardiographic features, provide additional prognostic information. Following validation in large prospective multicenter cohort studies, incorporating these readily identifiable variables into routine clinical assessment may improve stroke risk stratification, facilitate individualized preventive strategies, and ultimately reduce stroke-related morbidity and mortality.

## Conclusions

In this study, IS occurred in a considerable proportion of patients with non-valvular AF. Advanced age, previous stroke/TIA, vascular disease, smoking, chronic kidney disease, inadequate anticoagulation, left atrial enlargement, reduced LVEF, and LAA thrombus or SEC were identified as independent predictors of IS. These findings suggest that, in addition to the conventional CHA₂DS₂-VASc score, these clinical, treatment-related, and echocardiographic factors may provide additional prognostic information regarding stroke risk. However, validation of these findings in large prospective multicenter cohort studies is required before these factors can be incorporated into existing stroke risk assessment models. If validated, incorporating these readily identifiable factors into routine clinical risk assessment may improve stroke risk stratification, facilitate individualized preventive strategies, and ultimately reduce stroke-related morbidity and mortality, particularly in resource-limited healthcare settings.
